# Novel dihydroartemisinin dimer containing nitrogen atoms inhibits growth of endometrial cancer cells and may correlate with increasing intracellular peroxynitrite

**DOI:** 10.1038/s41598-019-52108-6

**Published:** 2019-10-29

**Authors:** Yan Zhu, Christian Klausen, Jieyun Zhou, Xiangjie Guo, Yu Zhang, Hua Zhu, Zhao Li, Jung-Chien Cheng, Shuwu Xie, Wenjie Yang, Ying Li, Peter C. K. Leung

**Affiliations:** 1 Laboratory of Reproductive Pharmacology, Shanghai Institute of Planned Parenthood Research; Key Lab. of Reproduction Regulation of NPFPC, SIPPR, IRD, Fudan University, Shanghai, 200032 China; 2 Department of Obstetrics and Gynaecology, BC Children’s Hospital Research Institute, University of British Columbia, Vancouver, British Columbia, V5Z 4H4 Canada; 3 Shanghai Institute of Materia Medica, Chinese Academy of Sciences, Shanghai, 201203 China

**Keywords:** Pharmacodynamics, Drug development

## Abstract

In the present study, a novel dimer, SM1044, selected from a series of dihydroartemisinin (DHA) derivatives containing nitrogen atoms comprising simple aliphatic amine linkers, showed strong growth inhibition in six types of human endometrial cancer (EC) cells, with half maximal inhibitory concentration (IC_50_) and 95% confidence interval (CI) < 3.6 (1.16~11.23) μM. SM1044 evoked apoptosis and activated caspase-3, −8 and −9 in a concentration- and time-dependent manner, and these effects were manifested early in RL95-2 compared to KLE cells, possibly correlated with the induction of intracellular ONOO^−^. Catalase and uric acid attenuated the growth inhibitory effects of SM1044 on EC cells, but sodium pyruvate did not. *In vivo*, the average xenograft tumour growth inhibition rates ranged from 35.8% to 49.9%, respectively, after 2.5 and 5.0 mg/kg SM1044 intraperitoneal treatment, and no obvious behavioural and histopathological abnormalities were observed in SM1044-treated mice in this context. SM1044 predominantly accumulated in the uteri of mice after a single injection. SM1044 displayed efficacy as a tumour suppressor with distinct mechanism of action and unique tissue distribution, properties that distinguish it from other artemisinin analogues. Our findings provide a new clue for artemisinin analogue against cancer.

## Introduction

Endometrial cancer (EC) is the fifth most common cancer in women^1,2^. Over the last decade, while cancer incidence rates have declined in the United States, the occurrence of EC has increased and is anticipated to continue this trend^3^. This pattern is also evident in China where the occurrence of EC has increased rapidly, showing the highest incidence of malignant tumour affecting females in big cities since 2008^4^. EC is generally classified into two subtypes — type I EC is of endometrioid origin, and hormone-receptor positive, and type II is non-endometrioid, *TP53*-mutated and hormone-receptor negative and associated with a higher risk of metastasis and a poor prognosis^1,5^. Some of the difficulties of EC therapy include a low response rate and high toxicity of existing drugs. For instance, the overall response rate of medroxyprogesterone for advanced or recurrent EC is approximately 25%^6^, and other medications are lower than 31%^1^. Chemotherapy after surgery did not demonstrate significant improvement in 5 year overall survival, progression-free survival and relapse, and also induced toxic effects^1^. The latter was evident in on-going mTOR inhibitor therapy that showed toxicity, including asymptomatic mucositis and grade 3 pneumonitis in phase II trials^7^. Consequently, there still exists a requirement for developing novel drugs towards EC adjuvant therapy.

Artemisinin (ART) is a widely used antimalarial medicine. Artemisinin-based combination therapies (ACT) are recommended by World Health Organization (WHO) for the treatment of malaria of children and adults (except pregnant women in their first trimester) and regarded as highly effective and well tolerated and substantially decrease morbidity and mortality from malaria^8^. Artemisinin and its derivatives are also being developed for treatment of cancer. The rationale is that cancer cells contain high concentration of free iron, similar to the malaria parasites^9^. Ever since artesunate, one of ART derivaties, was demonstrated to exert significant antitumor activity in 55 tumor cells^10^, diverse dimers, trimers and hybrids of ART derivatives have been synthesised, and some of them showed better bioavailability and bioactivity than ART in suppressing proliferation of certain cancer cells.

The safety and efficacy of long-term administering artemisinin derivatives have been evaluated in animals^11,12^ and in several clinical trials, including ten cervical carcinoma patients (stage III or IV) were treated with artenimol-R (100–200 mg) for 28 days in Ivory Coast^13^, twenty-three colorectal carcinoma patients were orally treated with 200 mg artesunate for 14 days in the UK^14^ and twenty-three metastatic breast cancer patients daily oral administration of artesunate (100–200 mg) for 4 ± 1 weeks in Germany. In animal experiment, part of animals did not show any sign of toxicity or adverse effects, while the other part of animals developed transient fever and haematological/gastrointestinal toxicity^11^ and histological lesions^12^, but these changes were restored in the recovery experiment^12^. In clinical trials, reduction of clinical symptoms of advanced carcinoma and tolerable adverse events (transient ‘flu-like syndrome headache and abdominal pain) were observed^13^, and only a few of patients among the three clinical trials were reported to experience severe adverse events (leucopenia, neutropenia, asthenia, anemia) possibly related to ART^15,16^. Despite limitations of a small study size, these encouraging results enhance our confidence to develop artemisinin derivatives for the treatment of cancer.

ART possesses the structure of sesquiterpene trioxane lactone. While the endoperoxide moiety of artemisinin was deemed crucial for its activity, certain synthesised compounds containing peroxy groups by themselves showed poor bioactivity, but extending the length of linker “C-O-C-” increased its bioactivity. Therefore, peroxy group was an essential, but not a sufficient factor^17^, and introducing an amino group into the artemisinin molecule enhances the water solubility of its derivatives and improves their bioavailability^18^. Moreover, the size and number of carbon atoms or spatial position of linkers likely play an important role and correlate to anticancer activity^19–22^. Therefore, we prepared a series of acetal 12β(C–O)-type dimeric DHA derivatives with varying lengths of the linker chain. In contrast to other linkers previously reported^19,20,23,24^, we synthesised nitrogen atom-containing linker groups of the dimers.

The aim of the study was to identify a candidate compound for anti-EC treatment from a repertoire of novel dimers and explore its possible mechanism of action. Since neurotoxicity related to long-term (>28 days) and high dose treatments has been reported in experimental animals^25,26^, neuron cells derived from animals could be a sensitive surrogate for assessing bioactivity of artemisinin analogues. Accordingly, we firstly evaluated the inhibitory activity of 10 novel dimers by using the most widely used rat PC12 neuronal cells^27^, and also a cardiomyocyte H9c2(2-1) cell line since cardiotoxicity was reported in artemisinin treated animals^25^. Subsequently, one of the dimers, SM1044, exhibiting the strongest activity was selected for additional tests in several human EC cell types, and its efficacy *in vivo* was confirmed in a Balb/c nude mouse bearing a tumour xenograft. Preliminary safety assessment was performed via histopathological examination, and tissue distribution of SM1044 was explored to reveal its potential target organ. Moreover, we investigated the time-course effect of SM1044 on the levels of hydrogen peroxide (H_2_O_2_), peroxynitrite (ONOO^−^), and apoptosis-related protein. Additionally, we analysed the association between molecular structure of the dimers and its inhibitory activity to enable structure modification of artemisinin analogues for boosting their tumour-suppression properties.

## Results

### Bioactivity of novel dimers and its relationship to linker groups

**Figure 1:**
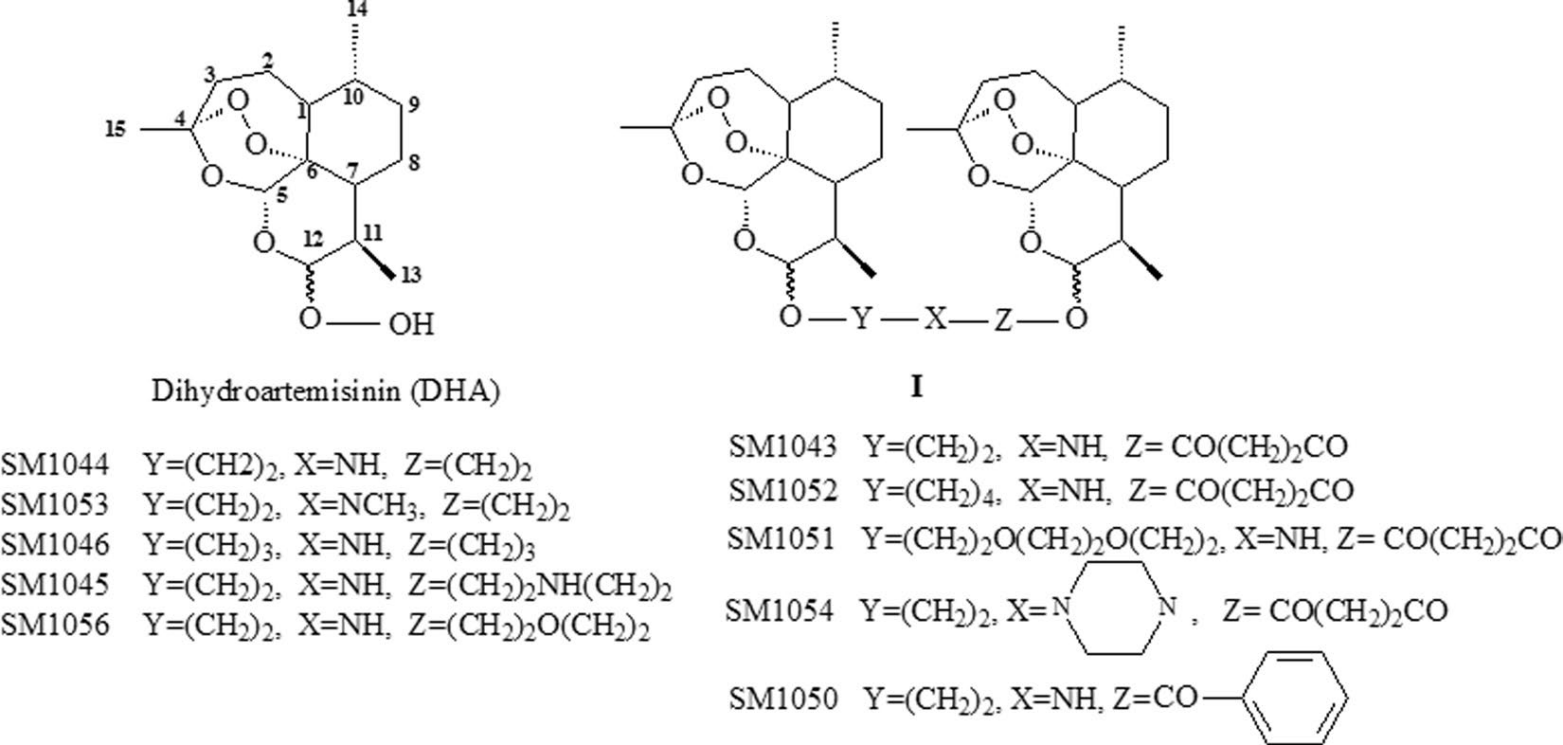
General structure of dimeric derivatives (**I**) and its parent drug, dihydroartemisinin (DHA).

Figure 1 shows the general structure of the dimers (**I**). Various derivatives were formed when linker chain, -Y-, -X- and -Z-, were replaced by different groups. Overall, the dimers demonstrated stronger inhibitory activity in PC12 cells than in H9c2(2-1) cells. Moreover, four of the dimers, SM1044, SM1045, SM1046 and SM1056, whose linkers merely contained aliphatic amine groups, displayed more potent inhibitory activity than the amide-containing dimers, SM1043, SM1050, SM1051, SM1052 and SM1054. Two arteether molecules of SM1044 that were connected via diethylamine groups, showed the strongest inhibitory activity of all the dimers tested, with an IC_50_ lower by 8.3 fold in PC12 cells and 10 fold in H9c2(2-1) cells compared to dihydroartemisinin (DHA) (Table 1, *P* < 0.05).

**Table 1:** Inhibitory activity of dimeric derivatives of DHA containing nitrogen atoms in PC12 and H9c2(2-1) cells after treatment for 48 h.

Compounds	Inhibitory Potency IC_50_ (95%CI) μM	
	PC-12 cells	H9c2(2-1) cells
DHA	9.54^*^ (0.55–28.83)	71.23^*^ (0.42–11945)
SM1044	1.15^#^ (0.15–8.72)	6.80^#^ (0.43~107.5)
SM1045	3.64 (0.48–27.67)	6.56^#^ (0.31~140.6)
SM1046	3.37 (0.38–29.54)	6.86^#^ (0.37~127.0)
SM1053	16.92^*^ (3.51–81.62)	14.68 (1.33~162.1)
SM1056	3.98 (0.55–28.83)	5.97^#^ (0.57–62.45)
SM1043	17.58^*^ (0.94–329.8)	173.4^*^ (4.75~6332)
SM1050	18.93^*^ (10.57–33.88)	31.22 (15.61~62.47)
SM1052	40.12^*^ (1.22–1316)	>700^*#^ (very wide)
SM1051	11.62^*^ (1.29–104.5)	46.99 (2.20 ~ 1001)
SM1054	8.48^*^ (1.23–58.69)	28.04 (2.35~334.5)

Data were presented as IC_50_ value (μM) with 95% confidence interval (95% CI). Experiments were performed in triplicate. *P < 0.05 *vs* SM1044 treatment. #*P* < 0.05 *vs* DHA treatment.

The inhibitory activity of SM1053, a dimer with its secondary amines replaced by methylamines, was approximately 16 fold lower than that of SM1044 (Table 1, *P* < 0.05), since compared to secondary ammonium, tertiary ammonium displays lower water solubility.

The length of linker chain was demonstrated to affect bioactivity in dimers with similar type of linkers. SM1045 and SM1046 consisting of diethyl ethylenediamine and dipropyl amine linkers, respectively, demonstrated 3 fold lower inhibitory activity in PC12 cells, compared to SM1044 which has a shorter linker than that of SM1045 and SM1046, Similarly, among dimers with amide groups, SM1052 containing butyl groups in the linker demonstrated 2.3 times lower inhibitory activity compared to that of SM1043, with has a longer linker (Table 1).

However, bioactivity was not enhanced by replacing one of acetyl with phenyl groups in the linker, such as SM1050, or adding more heteroatoms to the linker, such as SM1056 and SM1051, or changing the secondary amines to piperazine, such as SM1054. These dimers displayed lower inhibitory activity compared to SM1044 (Table 1, *P* < 0.05).

### *In vitro* inhibitory activity of SM1044 in human endometrial cancer cells

Due to its potent inhibitory activity in PC12 and H9c2(2-1) cells, SM1044 was used for further evaluation of its inhibitory activity in six human EC cells. As Table 2 shows, we found that the IC_50_ (95%CI) of SM1044 was < 3.60 (1.16~11.23) μM in both type I and type II EC cells. In RL95-2, HEC-1-A and AN3CA cells, the lowest IC_50_ of SM1044 was noted at 6 and 12 h post-treatment (*P* < 0.05, Table 2). SM1044 exhibited nearly equivalent suppression tested without any significant differences in KLE, HEC-1-B and HEC-50 cells with similar IC_50_ values at all time-points (Table 2, *P* > 0.05). In contrast, DHA demonstrated a 13 fold lower inhibitory potency than SM1044 in all tested cells, at the time-point of 12 h (*P* < 0.05, Table 2).

**Table 2:** Time-course effects of SM1044 and DHA on cell viability in six types of human endometrial cancer cells. Data were presented as IC_50_ value (μM) with 95% confidence interval (95% CI). Experiments were performed in triplicate each time and independently repeated three times with different passages of cells.

Tested compounds	Cell Line	Inhibitory Potency IC_50_ (95%CI) μM				
		3 h	6 h	12 h	24 h	48 h
SM044	RL95-2	2.06^b^ (0.39~10.78)	0.69^a,d^ (0.24~2.01)	1.14 (0.27~4.77)	2.13^b^ (0.98~4.62)	1.05 (0.23~4.84)
	KLE	2.50 (1.38~4.51)	1.16 (0.32~4.22)	1.52 (0.55~ 4.22)	2.43 (0.42~14.06)	1.78 (0.43~7.37)
	HEC-50	1.75 (0.28~11.10)	1.82 (0.29~11.47)	1.34 (0.35~5.08)	1.95 (0.79~4.77)	1.41 (0.67~2.95)
	HEC-1-A	3.60^c^ (1.16~11.23)	1.39 (0.35~5.52)	1.21^a^ (0.44~3.31)	2.73 (0.48~15.39)	2.21 (0.85~5.73)
	HEC-1-B	1.95 (0.40~9.56)	1.80 (0.34~9.56)	2.00 (0.43~9.19)	1.84 (0.57~5.87)	1.41 (0.38~5.24)
	AN3CA	3.22^b^ (0.70~14.76)	1.14^a^ (0.31~4.140)	1.87 (0.53~6.57)	2.62 (0.71~9.74)	1.46 (0.25~8.53)
DHA	RL95-2		59.74^*^ (10.51~339.6)	36.65* (18.17 ~73.95)		
	KLE		20.29* (5.23~78.82)	19.95* (17.98~22.13)		
	HEC-50		61.14* (40.51~92.27)	18.93* (2.88~124.6)		
	HEC-1-A		39.34* (0.42~ 3668)	26.88* (0.34 ~2143)		
	HEC-1-B		77.02* (5.12~ 1158)	74.85* (56.53~99.09)		
	AN3CA		82.37* (17.40~390.0)	44.29* (6.06~323.4)		

^a^*P* < 0.05 vs SM1044 treatment for 3 h. ^b^*P* < 0.05 vs SM1044 treatment for 6 h. ^c^*P* < 0.05 vs SM1044 treatment for 12 h. ^d^*P* < 0.05 vs SM1044 treatment for 24 h. *P < 0.05 vs SM1044 treatment at all time-point. Comparisons were conducted in the same cell lines.

### SM1044 induced apoptosis and expression of cleaved caspase-3, −8 and −9 in RL95-2 and KLE cells

**Figure 2:**
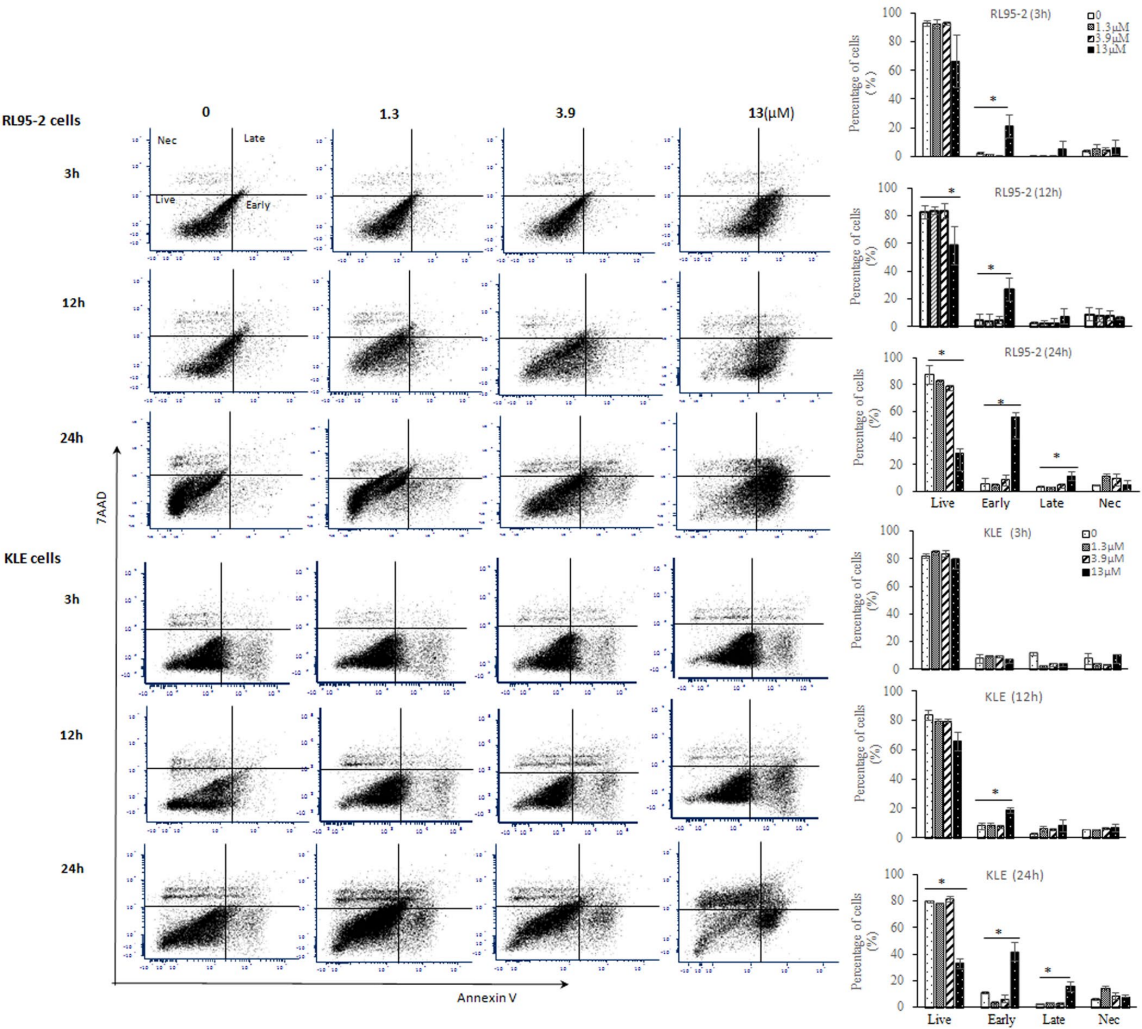
Time-course effects of SM1044 induced apoptosis in RL95-2 and KLE cells. Percentages of live, early-stage apoptosis, late-stage apoptosis and necrotic cells were determined by flow cytometry with Annexin V and 7-AAD staining after SM1044 treatment for 3, 12 and 24 h at the concentration of 0 (DMSO), 1.3, 3.9 or 13 μM. Bars represent as means ± SD of three independent samples. Live represents the percentage of live cells after treatment, and Early represents early-stage apoptosis, and Late represents late-stage apoptosis, and Nec represents necrotic cells. *P < 0.05 *vs* the percentage of each cell type in control which was treated with vehicle (DMSO).

Based on the strong inhibitory activity of SM1044 in human EC cells *in vitro*, RL95-2 (type I) and KLE (type II) cells were chosen for further investigations. The percentage of early-stage apoptotic cells, detected by FACS, was markedly increased 3 h after SM1044 treatment in RL95-2 cells (*P* < 0.05, Fig. 2). A significant increase in percentage of early-stage apoptotic cells was observed 12 h after SM1044 treatment in KLE cells and at 24 h, the percentage of both early-stage and late-stage apoptotic cells increased significantly compared to the control (*P* < 0.05, Fig. 2).

**Figure 3:**
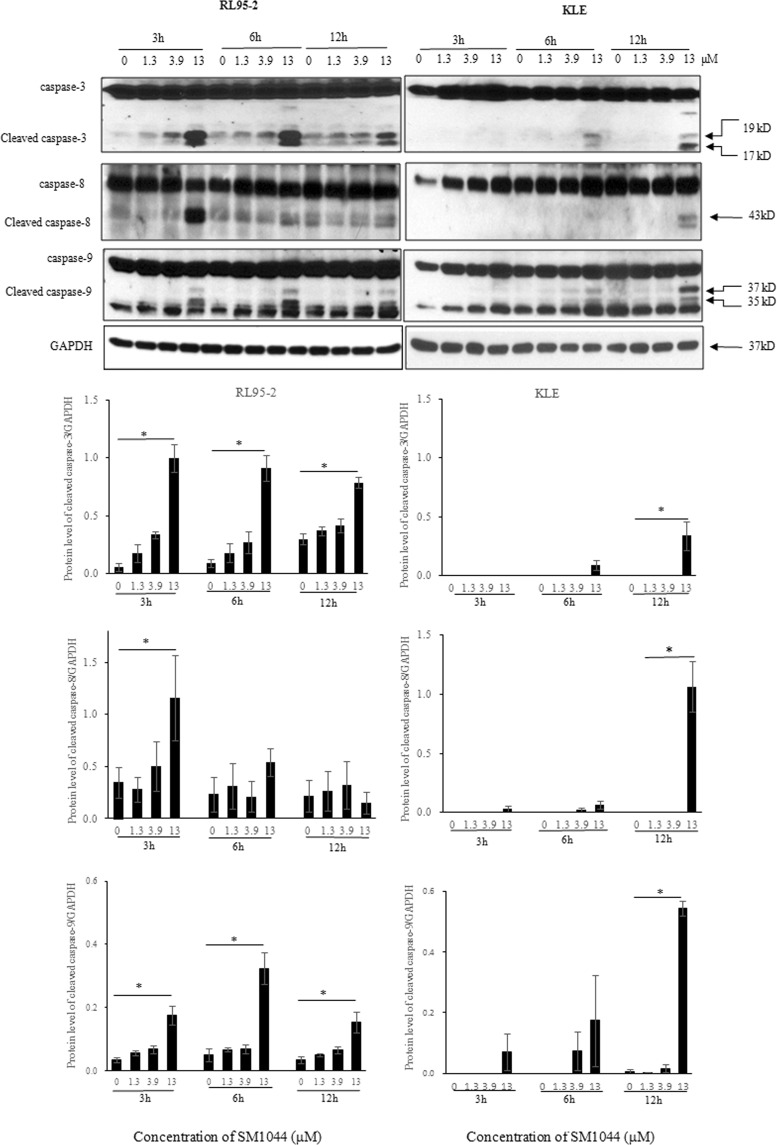
Time-course effects of SM1044 on protein levels of activated caspase-3, 8, and 9 in RL95-2 and KLE cells, as determined by western blotting (quantified data were normalised to GAPDH) after both cells were treated with DMSO (control) or graded concentrations of SM1044 (1.3, 3.9 or 13 μM) for 3, 6 and 12 h, respectively. Data were presented as mean ± SD of three independent samples. *P < 0.05 *vs* control.

Since caspase activation is a hallmark and mediator of apoptosis, the expression of several activated caspase-signalling proteins was measured using western blotting. In both RL95-2 (type I) and KLE (type II) cells tested, no remarkable changes in the expression of total caspase were observed after SM1044 treatment. However, in RL95-2 cells, the expression of cleaved caspase (CC)−3, the most important executioner caspase, and CC-8 and CC-9, key enzymes in the extrinsic and intrinsic apoptosis pathway, respectively, were simultaneously expressed in a concentration-dependent manner after SM1044 treatment for 3 h (*P* < 0.05, Fig. 3). Expression of CC-9 and CC-3 were observed after SM1044 treatment for 6 h in KLE cells, and CC-3 and CC-8 showed a concentration-dependent increase at 12 h (*P* < 0.05, Fig. 3). SM1044 induced apoptosis in both cells equally after treatment for 12 h. These findings indicate that the activation of apoptosis appeared earlier and more rapidly in RL95-2 compared to KLE cells.

### Effects of SM1044 on the levels of H_2_O_2_ and ONOO^−^ in RL95-2 and KLE cells

To investigate the possible mechanism of action of SM1044-induced apoptosis, we measured relative levels of H_2_O_2_ and ONOO^−^/•OH in RL95-2 (type I) and KLE (type II) cells after SM1044 treatment in the presence or absence of catalase, uric acid and sodium pyruvate, which are the protector of ROS, the scavenger of ONOO^−^ and H_2_O_2_, respectively.

**Figure 4:**
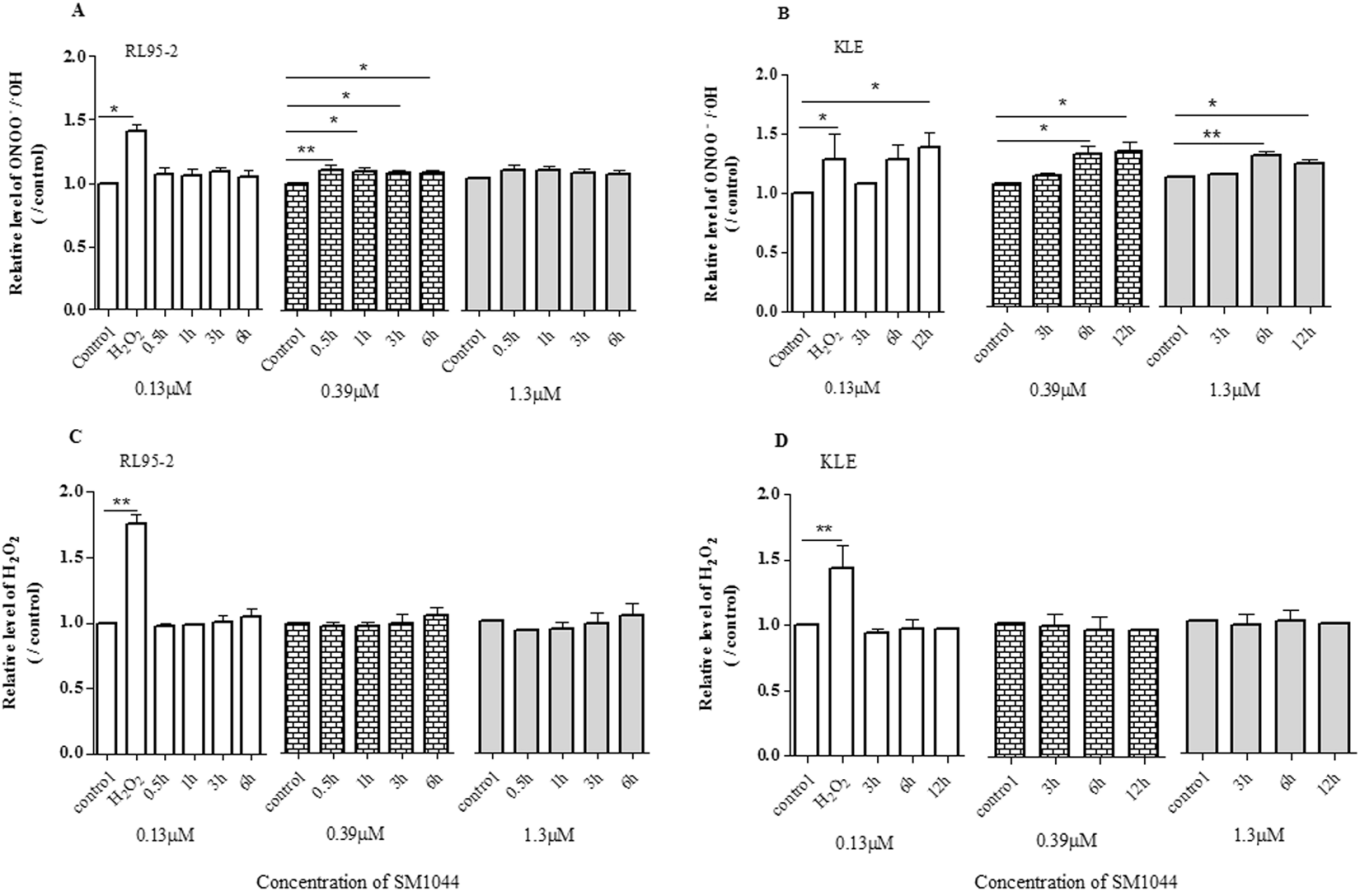
Effect of SM1044 on intracellular level of peroxynitrite (ONOO^−^) and hydrogen peroxide (H_2_O_2_). The cells treated with DMSO served as the negative control. Results were presented as relative level of ONOO− or H_2_O_2_, expressed as the ratio of fluorescence intensity of treated cells divided by that of negative control cells and expressed as fold increase compared with the control. Data were presented as mean ± SD of triplicate with three independent samples. *P < 0.05, **P < 0.01 *vs* the relative level of the control.

In RL95-2 cells, ONOO^−^/•OH increased shortly after SM1044 treatment for 30 min and lasted after treatment for 6 h, with cells simultaneously undergoing apoptosis (*P* < 0.05, Fig. 4A). Increased level of ONOO^−^/•OH was observed in KLE cells exposed to SM1044 for more than 6 h (*P* < 0.05, Fig. 4B), consistent with the appearance of apoptosis. However, there was no significant change in the relative level of H_2_O_2_ in both RL95-2 and KLE cells after SM1044 treatment compared with that of control (*P* > 0.05, Fig. 4C,D).

**Figure 5:**
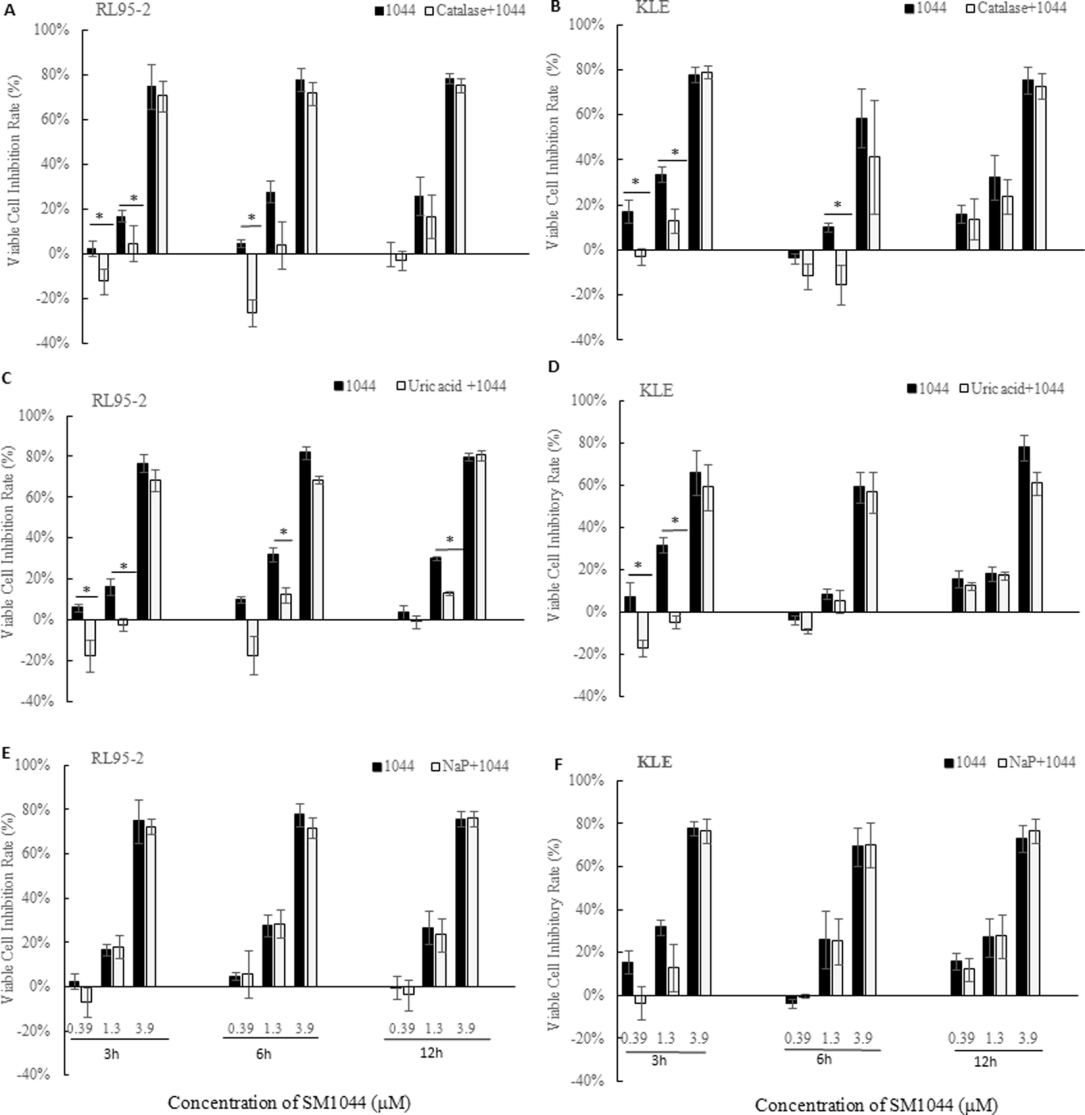
Effect of catalase, uric acid and sodium pyruvate (NaP) on cell growth inhibition in RL95-2 and KLE cells in the presence of SM1044, as assessed by MTT. Cells were pretreated with the scavengers, catalase (0.05 mg/ml), uric acid (100 μM) and NaP (10 mM), for 20 min prior to treatment with SM1044. Data were shown as viable cell inhibition rate and presented as mean ± SD of triplicate with three independent samples. *P < 0.05 *vs* the inhibition rate of the cells treated with SM1044 alone.

Furthermore, pretreating RL95-2 and KLE cells with catalase (0.05 mg/ml) and uric acid (100 μM) reversed or reduced the cell growth inhibition induced by SM1044. A significant difference in reversing growth inhibition was observed in both RL95-2 and KLE cells at low concentrations of SM1044 (0.39 and 1.3 μM) treatment for 3 and 6 h, but not at higher concentration of SM1044 (3.9 μM) treated cells (*P* < 0.05, Fig. 5A–D). This indicated that catalase and uric acid could antagonise the inhibitory effect induced by SM1044 and protect the cells from SM1044-induced cell damage up to a certain extent. In contrast, pretreating both cells with sodium pyruvate (10 mM) did not significantly attenuate the cell growth inhibition mediated by SM1044 (*P* > 0.05, Fig. 5E,F)

### Suppressive efficacy of SM1044 on the growth of RL95-2 xenograft tumour *in vivo*

**Figure 6:**
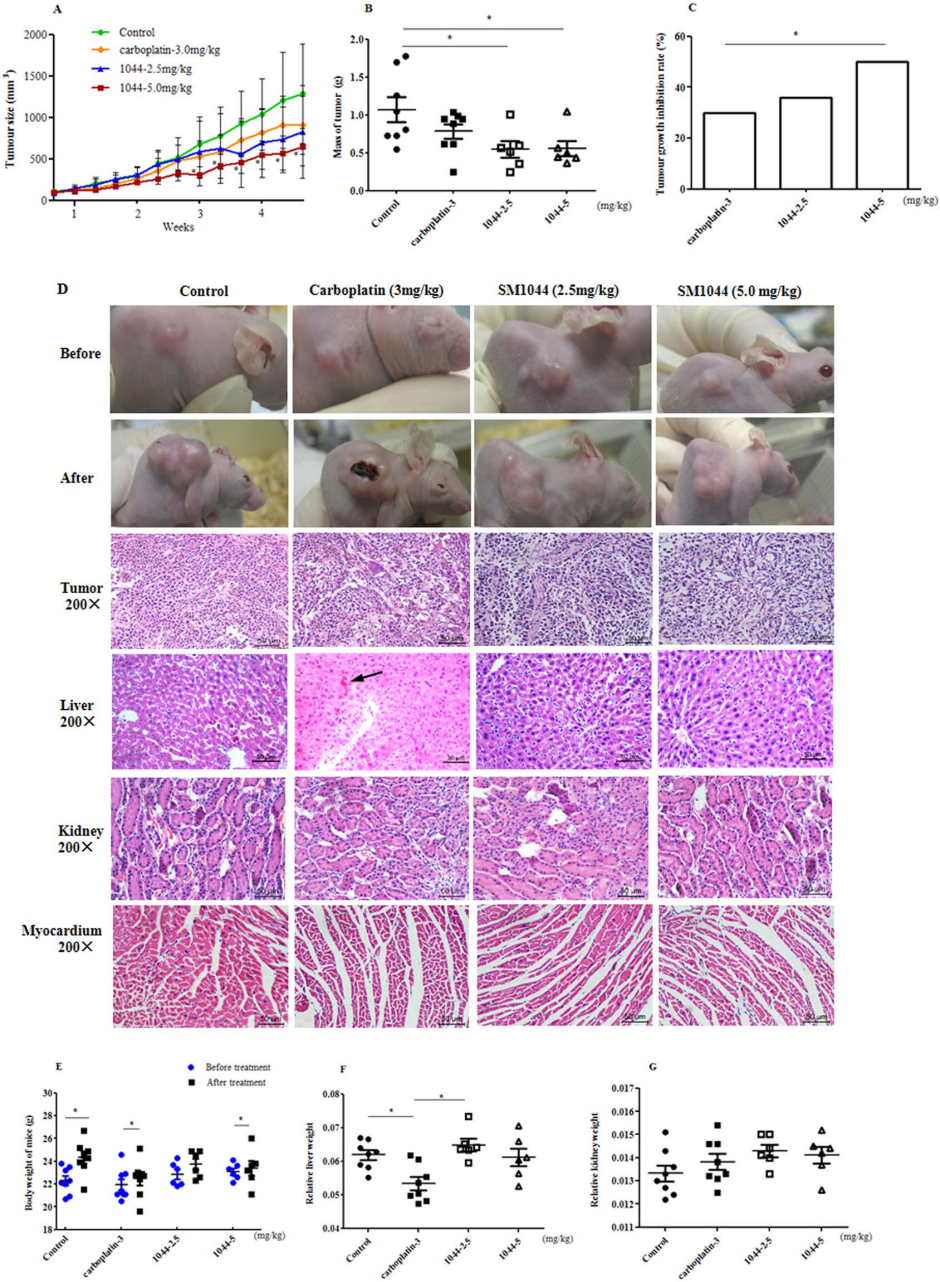
Effect of SM1044 on xenograft tumour in BALB/c athymic nude mice bearing RL95-2 cells before and after 4 weeks of treatment. (**A**) Growth curves of tumour size during 4 weeks treatment. (**B**) Mass of tumour after animals were treated with 0.5%CMC-Na (control) (n = 8), carboplatin (3.0 mg/kg, n = 8) and SM1044 (2.5 and 5.0 mg/kg, n = 6, respectively) for 4 weeks. (**C**) Tumour growth inhibition rate (%) after carboplatin and SM1044 treatment, against control group. (**D**) Gross morphology and histopathology before and after treatment with SM1044 and carboplatin (H&E, 200 × magnification). (**E**) Body weight of animals before and after treatment. (**F**) Relative liver weights of animals after treatment. (**G**) Relative kidney weights of animals after treatment. Data were presented as mean ± SD of independent samples. *P < 0.05 *vs* control (treated with solvent). ^a^*P* < 0.05 *vs* carboplatin treatment. ^b^*P < *0.05 *vs* 5.0 mg/kg SM1044 treatment. ^#^*P* < 0.05 *vs* prior to treatment. Arrowhead indicates location of haemorrhage in the liver.

The safety and efficacy of SM1044 were evaluated in a xenograft tumour model of Balb/C nude mice bearing RL95-2 cells, since our experiments showed these cells to be more sensitive to SM1044 than KLE cells. Two weeks after 28 nude were inoculated with RL95-2 cells mice, solid xenografts measuring up to 100 mm^3^ were observed in all mice. Prior to treatment, there were no remarkable differences in tumour sizes among all groups (*P* > 0.05, Fig. 6A). At the end of 4 weeks of treatment, there was a pronounced decrease in size and mass of tumour in animals treated with SM1044 (*P < *0.05, Fig. 6A,B), compared with the control group. The average tumour growth inhibition rate was 35.77% and 49.92% in 2.5 and 5.0 mg/kg SM1044 treatment groups, respectively. In contrast, tumour growth inhibition rate in 3.0 mg/kg carboplatin-treated mice was 29.76%, which was lower than that in SM1044 (*P < *0.05, Fig. 6C).

Gross morphological assessment of the xenografts showed a cauliflower shaped, grey, solid, irregular circle or oval entity (Fig. 6D). Pathologically, the xenografts demonstrated features of human endometrial cancer, including clear nest of cancer with boundaries, enlarged and darkly stained nuclei, irregular nuclear size, and closely lined cells. In the treated groups, overall the xenograft tumour appeared loose with increased cytoplasmic vacuolisation (Fig. 6D). No death occurred in mice during treatment, no abnormal behaviour and physiological signs were observed, and also no tumour metastasis were found; an increase in body weight was observed (*P* < 0.05, Fig. 6E). While there was a significant reduction in relative liver weight in carboplatin-treated mice (*P* < 0.05, Fig. 6F), compared to the control, no difference in relative liver weight was observed in animals treated with SM1044 (*P* > 0.05, Fig. 6F). Consistent with this observation, pathological haemorrhage of liver was observed in five of six carboplatin treated- and in one of six solvent and SM1044 (2.5 mg/kg) treatment mice, respectively, without abnormalities in the 5.0 mg/kg SM1044 treated mice (Fig. 6D). No remarkable pathological abnormalities of kidney and myocardium were observed in the tested animals (Fig. 6D,G).

### Preliminary tissue distribution of SM1044

**Figure 7:**
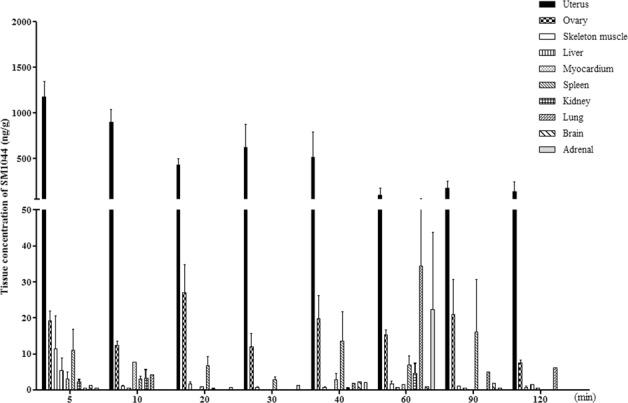
Time-course of tissue distribution of SM1044 after 5 mg/kg single intraperitoneal administration to female mice (n = 6). Tissue samples were collected and processed for HPLC/MS determination. Each data point represents mean ± SD.

Additionally, we preliminarily explored the tissue distribution of SM1044 to reveal its potential target organ in mice. SM1044 was rapidly distributed into tissues after i.p injection and accumulated more in uterus than the other test tissues. The maximal concentration of SM1044 was detected in uterus (1177.70 ± 416.79 ng/g) after dosing for 5 min and then gradually declined and the average concentration of six mice maintained at 139.78 ± 253.72 ng /g after dosing 120 min. SM1044 was also detected in ovary and spleen during most of time point assayed. In other test tissues, however, including skeleton muscles, lung, liver, kidney, myocardium, brain and adrenal gland, the concentration of SM1044 was extremely low and even not detectable at some of timepoints (Fig. 7).

## Discussion

We herein describe SM1044, a novel DHA dimer containing nitrogen atoms, with antitumor properties. SMI1044 exhibited strong suppression of growth of human EC cells and xenograft tumor in mice through the induction of cellular apoptosis likely associating with reactive nitrogen species (RNS). Type I EC cells showed unique sensitivity in response to SM1044 treatment and *in vivo*, this molecule displayed unique tissue distribution.

The inhibitory activity of the novel dimeric derivatives correlated with the chemical properties and also to the length of linker chain to a certain extent. Dimers containing merely aliphatic amines generally displayed stronger inhibitory activity than those containing amide groups, and imparting amide groups into the linker led to poor bioactivity. Moderately extending the length of linker by replacing amines with methylamine groups or introducing more heteroatoms into linker chain failed to improve the inhibitory activity. Our finding is consistent with that reported by Jung *et al*.^19^, who reported that the antitumor activities of dimers were dependent on the length of the linker in non-acetal deoxoartemisinin derivatives, and longer linker would result in poor antitumor activity.

Furthermore, we found that SM1044 exhibited strong inhibitory activity on different types of EC cells. RL95-2 and HEC-1-A are oestrogen receptor-positive^28,29^, whereas KLE, HEC-50, HEC-1-B and AN3CA are oestrogen unresponsive EC cells^30–32^. Among these cell types, HEC-1-A, HEC-1-B, KLE, HEC-50 and AN3CA cells have p53 mutations^33–35^, HEC-1-B and AN3CA bear Kras and fibroblast growth factor receptor 2 (FGFR2) mutations respectively, which are associated with drug-resistance^36,37^. Accordingly, our results showed that SM1044 is a potent inhibitor, suppressing growth of not only oestrogen receptor responsive (type I) cells, but also of type II cells with gene mutations and drug resistance. SM1044 was previously reported to induce cell cycle arrest in G(0)/G(1) phase in a leukemic cell line^38^. Here, we found SM1044 induced apoptosis in prototypical type I (RL95-2) and type II (KLE) cells which was associated with activation of intrinsic and extrinsic caspases. Interestingly, SM1044 exhibited a time-dependent effect on apoptosis since there was rapid apoptosis in RL95-2 cells compared to KLE cells showing that type I EC cells were more sensitive to SM1044-induced apoptosis than type II EC cells.

It is generally considered that one of the mechanisms by which artemisinin and its analogues targets parasite and cancer cells is via cleavage of the endoperoxide bridge to generate free radicals, such as reactive oxygen species (ROS) and other free radicals, leading to subsequent oxidative damage and induction of cell apoptosis and necrosis^39,40^. Indeed, ROS-induced apoptosis by artemisinin analogues was found in epithelial, mesenchymal or haematopoietic tumour cells^16^. Accordingly, we used a fluorescent dye, 5-(and-6)-chloromethyl-2,7-dichlorodihydrofluorescein diacetate acetyl ester (CM-H_2_DCFDA) to examine the production of ROS in a preliminary test, but did not observe distinct alteration in fluorescence signal between the control and SM1044 treated cells (data not shown). Since H_2_O_2_ is a reactive oxygen metabolic byproduct that can be easily quantified, we further measured the production of H_2_O_2_ in this study, but found no significant difference in intracellular H_2_O_2_ after SM1044 treatment. Moreover, pretreating EC cells with sodium pyruvate, a strong scavenger of H_2_O_2_, could not nullify the inhibitory effect mediated by SM1044 on cell damage. Taken together, these results suggest that it is less likely for SM1044 to induce apoptosis of EC cells via H_2_O_2_. However, pretreating both RL95-2 and KLE cells with catalase significantly reduced the cell growth inhibition mediated by SM1044. Since catalase not only catalyses the decomposition of H_2_O_2_ protecting the cell from oxidative damage by ROS, but also scavenges peroxynitrite (ONOO^−^)^41^, we therefore presume that ONOO^−^ may play a role in SM1044-mediated cell death.

ONOO^−^ is a reactive nitrogen species (RNS) that reacts with key components of mitochondria to cause oxidative damage^42^; low concentrations trigger cell apoptosis, whereas higher concentrations induce necrosis^43^. Peroxynitrite decomposes to form nitrogen dioxide and hydroxyl radical (•OH), which are major *in vivo* toxins^44^. We assayed for hydroxyl radical/peroxynitrite and found that SM1044 significantly increased the level of ONOO^−^/•OH in both RL95-2 and KLE cells, and the elevation is nearly synchronised with the appearance of apoptosis. Further, uric acid, the scavenger of ONOO^−^ could reverse the inhibitory effect of SM1044 on EC cells. This suggests ONOO^−^/•OH may be associated with SM1044-mediated cell death. DMSO, a potent scavenger of hydroxyl radical (•OH) and the solvent of SM1044, did not have an effect. According to Pou *et al*.^43^, the yield of •OH produced by ONOO^−^ was as low as about l-4%, and therefore, the cytotoxicity mediated by peroxynitrite might not correlate to the formation of •OH. Based on the evidence, it is plausible for apoptosis induced by SM1044 to be mediated by ONOO^−^, though a role of •OH cannot be rule out during this process and therefore warrants additional studies.

In addition, tissue distribution profile showed that SM1044 had unique features, and the uteri were primary target tissues of SM1044 after a single intraperitoneal injection in mice, with similar findings in rats (data not shown). It implies an opportunity for SM1044 which could be used in gynaecological disease treatment. Since the concentration of SM1044 was low in brain and myocardium and meanwhile no obvious behavioural and histological changes were observed in mice, we presume that SM1044 may not exert serious damage to brain and myocardium in the test doses.

Our earlier studies showed that nomegestrol acetate and medroxyprogesterone displayed strong inhibitory effect on xenograft tumour in mice but the effective dose needed was high (up to 100 mg/kg)^45^. In this experiment, despite SM1044 showed efficacy in growth inhibition of xenografts in mice and caused fewer liver injuries than in carboplatin-treated mice, whether or not SM1044 would have superiority over hormonal and chemotherapy medicine is yet to be warrant. Due to lower doses, which merely equal to 0.3–0.6 mg/kg for human being were used in the experiment, the overall safety of SM1044 is yet to be further confirmed. Nontprasert A *et al*.^46^ reported that the 100% surviving mice with normal balance and gait was below 200 mg/kg orally administering DHA but significant neurological abnormalities occurred over 250 mg/kg/day. Deeken *et al*.^47^ reported that liver function damage and electrolyte disturbances were observed when artesunate were intravenously injected to patients more than the dose of 12 mg/kg. Accordingly, it should not exclude the possibility that the toxicity of SM1044 could appear with the increasing of the dose.

In summary, a novel dihydroartemisinin dimer, SM1044, was identified having capabilities against the growth of EC in the test circumstance. SM1044 mainly targeted the uterus and exhibited rapid and potent inhibitory actions targeting both hormone-receptor positive and non-hormone dependent EC cells, with type I EC cells showing more sensitivity compared to type II EC cells. Our findings provide a new clue for artemisinin analogue against cancer. However, there are limitations in the experiment. The role of peroxynitrite in SM1004-mediated apoptosis and studies on the metabolism of this novel dimer are yet to be thoroughly elaborated, and further larger scale trials are needed to provide more compelling evidence on efficacy and safety of this type of compounds in EC therapy.

## Materials and Methods

### Chemicals and reagents

Figure 1 shows the structures of SM1044 (maleate, C_38_H_59_NO_14_, molecular weight (MW) 753.8), SM1052 (C_38_H_59_NO_12_, MW 721.87), SM1043 (C_36_H_55_NO_12_, MW 693.8), SM1045 (maleate, C_36_H_60_N_2_O_10_, MW 680.9), SM1046 (maleate, C_40_H_63_NO_14_, MW 781.9), SM1050 (C_39_H_55_NO_11_, MW 713.9), SM1051 (C_40_H_63_NO_14_, MW 781.93), SM1052 (C_38_H_59_NO_12_, MW 721.81), SM1053 (maleate, C_39_H_61_NO_14_, MW 767.9), SM1054 (C_40_H_62_N_2_O_12_, MW762.93) and SM1056 (maleate, C_39_H_61_NO_14_, MW 797.9). All these compounds were synthesised according to a published patent^48^. The purity of all compounds were >95% and confirmed via melting point detection, ^1^HNMR spectra, IR spectra and elemental analysis. Dihydroartemisinin (DHA), dimethyl sulphoxide (DMSO), catalase (from bovine liver powder, suitable for cell culture, 2,000–5,000 units/mg protein), sodium pyruvate (NaP), uric acid and MTT (3-(4,5-Dimethylthiazol-2-yl)-2,5-diphenyltetrazolium bromide) were purchased from Sigma (Sigma-Aldrich, St. Louis, MO, USA). Carboplatin injection was purchased from Qilu pharmaceutical (Shandong province, China). Hydrogen peroxide/Peroxidase Detection Kit (Fluoro H_2_O_2_^TM^, Part t # 5016) and Hydroxyl radical/Peroxynitrite (ONOO-) Detection kit (HPF)(Part# 5020, Cat # FLHPF100-2) were purchased from Cell technology (Cell Technology Inc. Mountain View, CA, USA). Annexin V Apoptosis Detection Kit -PE and Accutase® Enzyme cell detachment medium were purchased from eBioscience Inc (San Diego, CA92121,USA). Caspase-8 (1C12) mouse mAb, caspase-3 antibody, cleaved caspase-3 (ASP175)(5A1E) rabbit mAb, and caspase-9 antibody (human specific) were purchased from CST (Cell Signaling Tech, Inc). GAPDH antibody (G-9) was purchased from Santa Cruz Biotechnology, Inc. (Santa Cruz, California, USA). The compounds tested were dissolved in DMSO and diluted to the desired concentration for *in vitro* experiments and in sterilised 0.5% sodium carboxymethylcellulose (CMC-Na, Sinopharm Chemical Reagent Co., Ltd, China) for *in vivo* studies.

### Cell lines and treatment

Rat pheochromocytoma (PC-12) and embryo myocardium H9c2(2-1) cells were purchased from Chinese Academy Science Cell Bank (Shanghai, China). RL95-2, HEC-1-A, HEC-1-B, KLE and AN3CA human endometrial cancer cells were purchased from the American Type Culture Collection (ATCC, Manassas, VA). HEC-50 cells, also known as HEC-50B, were obtained from the OVCARE Cell Bank (Vancouver, BC). PC-12 cells were cultured in RPMI1640 (Gibco, Life Technologies) supplemented with 10% fetal bovine serum (FBS; Gibco, Life Technologies). HEC-1-B and AN3CA cells were cultured in Minimal Essential Medium (MEM; Gibco, Life Technologies) supplemented with 10% FBS. H9c2(2-1), RL95-2, KLE and HEC-50 cells were cultured in Dulbecco’s Modified Eagle Medium/Nutrient Mixture F-12 1:1 (DMEM/F12; Gibco, Life Technologies) supplemented with 10% FBS. HEC-1-A cells were cultured in McCoy’s 5 A modified medium with 10% FBS. Cultures were maintained at 37 °C in a humidified atmosphere of 5% CO_2_ in air. Culture media were replaced every 2 or 3 days until the cells reached approximately 80% confluence, and then the cells were subcultured or treated with DMSO (as a control) or compound tested. The final concentration of DMSO in the culture media was 0.5% (v/v).

### Animals

Twenty-eight female athymic nude mice (BALB/C, nu/nu) and fifty-four ICR female mice were purchased from Sino-British Experiment Animal Co. (Shanghai, China) and bred under specific pathogen free (SPF) animal house at the Institute of Planned Parenthood Research (SIPPR). Animals were treated in compliance with guidelines of the Institutional Animal Care and Use Committee of SIPPR (Animal Experiment Ethics Approval No. 2016-6). Animals were housed four per cage under a 12-h/12-h light/dark cycle with free access to standard chow and sterilised tap water. Prior to the experiment, the mice were fed for one week’s adaption. Each animal was weighed once per week. At the end of the experiment, pentobarbital sodium was used for anaesthesia of the animals.

### Cell viability assays and IC50 calculation

MTT assay was used to assess the suppressive effects of the dimers and DHA on cell viability in rat PC12 and H9c2 (2-1) cells and human EC cells. Briefly, cells were cultured in 96-well plates and treated with DMSO or graded concentrations of DHA and target derivatives. MTT (final concentration of 0.5 mg/mL) was added at each time-point tested. A microplate reader (PerkinElmer Enspire® Victor 2 V Multilabel Reader) was used to measure optical density (OD) at 560 nm. Viable cell inhibition rate (%) was calculated using the following equation: [(1-OD of tested compound treatment cells/OD of control cells) × 100%] according to a previously described method^49^. Final results were presented as half maximal inhibitory concentration (IC_50_) with 95% confidence intervals (95% CI), which were calculated from a nonlinear regression model (curvefit) based on a sigmoidal curve of dose-response (viable cell inhibition rate), using GraphPad Prism 5 (GraphPad Software Inc, La Jolla, CA, USA). For growth curve analysis, the number of cells in each line was 10,000 each for PC12, H9c2 (2-1) and AN3CA, 5,000 each for RL95-2, KLE, HEC-1-A and HEC-1-B, and 2,500 for HEC-50.

MTT assay was also used to measure the antagonism of ROS/RNS scavengers against SM1044-induced cell growth inhibition. Briefly, catalase, sodium pyruvate (10 mM) and uric acid (100 μM) (ROS/RNS scavengers) hydrogen peroxide (H_2_O_2_) and peroxynitrite (ONOO-), respectively, were added to both RL95-2 and KLE cells for 20 mins, followed by treatment with SM1044 (final concentrations, 0.39, 1.3 and 3.9 μM) for varying time periods. Later, MTT was added and absorbance measured. Experiments were performed in triplicate each time and repeated independently three times with different passages of cells. Results were presented as viable cell inhibition rate (%) using the equation described above.

### Hydrogen Peroxide (H_2_O_2_) and Peroxynitrite (ONOO-) assay

Hydrogen Peroxide/Peroxidase Detection Kit (Fluoro H_2_O_2_^TM^) and Hydroxyl radical/Peroxynitrite Detection kit were used to assay the relative intracellular levels of H_2_O_2_ and ONOO^−^, respectively. Performances were conducted in accordance with the manufacturer’s instruction. Cells were seeded into 96-well plates at 1.5 × 10^4^ cells per well and cultured overnight. Wells were replaced with 200 μL of fresh medium and then the cells were treated with 1 μl of DMSO (negative control), 10 μM H_2_O_2_ (positive control) or SM1044 (final concentrations, 0.13, 0.39 or 1.3 μM). For the detection of H_2_O_2_, 50 μl of reaction cocktail was added to cells, incubated for 10 min at room temperature away from light, and then fluorescence intensity was measured in a multilabel plate reader (PerkinElmer Enspire ®2300 Multilabel Reader, Waltham, MA, USA) at excitation of 550 nm and emission of 595 nm at various time-points.

Detection of ONOO^−^ was performed according to the manufacturer’s instructions. Briefly, cells were rinsed with modified HBSS once, loaded with 2 μM of HPF (final concentration) and incubated for 60 minutes at 37 °C in the dark. Without washing, the cells were treated with 1 μl of DMSO or SM1044 (final concentrations, 0.13, 0.39 and 1.3 μM). 50 μM H_2_O_2_ was used as a positive control. Fluorescence intensity was measured on a multilabel plate reader (PerkinElmer Enspire ®2300 Multilabel Reader, Waltham, MA, USA) at excitation of 488 nm and emission of 515 nm at various time-points.

Experiments were performed in triplicate and were repeated independently three times with different passages of cells. The results were presented as relative level of ONOO^-^ or H_2_O_2_, expressed as the ratio of fluorescence intensity of SM1044 – or H_2_O_2_ – treated cells divided by DMSO treated cells.

### Flow cytometer assay (FACS)

Annexin V/7-AAD double staining kit was used to measure live, apoptotic (early and late-stage) and necrotic cells following treatment. RL95-2 and KLE cells were treated with 1 μl of DMSO (as control) or SM1044 (final concentrations, 1.3, 3.9 or 13.0 μM) for 3, 12 and 24 h. Cells were harvested and then performed according to the manufacturer’s instructions. Percentages of necrotic and apoptotic cells were measured using a BD FACS Canto Flow Cytometer (BD Inc, San Jose, CA, USA), and analysed with FCS Express 6 software (De Novo Software, Glendale, CA, USA). Experiments were independently repeated thrice and 15,000 cells were counted for each sample.

### Western blot analysis

Western blotting was used to evaluate the activity and the protein level of caspase-3, −8, and −9. Protein was quantified with the BCA Protein Assay (Bio-Rad Laboratories) and 50 μg were loaded onto 10% SDS-PAGE (30% acrylamide: bisacrylamide 29:1, Bio-Rad Laboratories) gels for electrophoresis and, then, transferred to PVDF membranes. Immuno-reactive bands were probed using enhanced chemiluminescent substrate or SuperSignal® West Femto maximum sensitivity substrate (Pierce, Thermo Fisher Scientific) and, then, exposed to X-ray film (Kodak, Rochester, NY, USA) and the image was acquired via scanning film. Primary antibodies against caspase-3, active caspase-3, caspase-8 and caspase-9 were diluted 1:1000. The primary GAPDH antibody was diluted 1:10,000. Densitometry analysis of target protein was performed using the Image-Pro Plus® Version 5.1.0 software (Media Cybernetics Inc.), with GAPDH as internal control for normalisation and expressed as relative fold change over the control.

### Xenograft assay

The xenograft nude mouse model bearing RL95-2 cells was established according to Morton CL *et al*.^50^ with a minor modification. RL95-2 cells were inoculated (2 × 10^7^ cells per mouse) subcutaneously into BALB/c nude mice (7~8 wk). The tumour size was calculated in accordance to the equation: V (mm^3^) = 1/2 × a × b^2^, in which *a* represents the longest diameter of tumour and *b* represents the shortest diameter. After the tumour size nealy reached 100 mm^3^ or larger, mice were randomly allotted into four groups and treatment was initiated. Group 1 served as the control, wherein animals were intraperitoneally treated with solvent. Group 2 mice were intraperitoneally administered carboplatin (3.0 mg/kg). Group 3 and 4 mice were intraperitoneally administered SM1044 2.5 and 5.0 mg/kg, respectively, once every other day, 3 times per week for consecutive 4 weeks. After 2 h last dosing, the mice were deeply anaesthetised and sacrificed with blood drawn and tumours and organs promptly removed and fixed in formalin solution.

The average tumour growth inhibition rate was used to evaluate the efficacy of the tested compounds and calculated via the formula: 100% × (V*c* − V*t*)/V*c*; V*c* and V*t* represents average volume of tumour of animals in control and SM1044 or carboplatin treatment, respectively. The relative weights of liver and kidney were calculated as the organ weight divided by the mouse body weight of mice.

### Histological examinations

Formalin-fixed xenograft tumour tissue, as well as liver, kidney, and heart of nude mice were embedded in paraffin and then sliced into 4-μm thick sections using a rotary microtome. Then, sections were stained with haematoxylin-eosin (H&E) for histopathological examination using light microscopy.

### Tissue kinetics of SM1044

Fifty-four ICR female mice (body weight 18-22 g) were used to investigate tissue distribution of SM1044. Animals were randomly divided into nine groups according to body weight and injected introperitoneally (i.p) with normal saline (as control) or SM1044 once at a dose of 5 mg/kg. After dosing, six mice per group were anaesthetised and sacrificed at designated time-points of 5, 10, 20, 30, 40, 60, 90 and 120 min, respectively. Tissues, including brain, liver, kidney, heart, lung, skeletal muscle, spleen, adrenal, ovary and uterus were promptly removed and homogenised with saline (1 ml/100 mg) using MT-30K homogeniser (Hangzhou MIU Instruments Co., Ltd, China). SM1044 was isolated from tissues by liquid-liquid extraction after adding diethyl ether. Artemether was used as internal standard working solution. Briefly, 10 μL of working solution of artemether and 1 mL of diethyl ether were added to 100 μL tissue homogenate buffer, vortexed, and then centrifuged (microfuge 22R centrifuge, Beckman Coulter) at 4°C. The organic layer (0.98 mL) was drawn and evaporated under a stream of nitrogen at 45 °C. Then, the dried residue was re-dissolved with 100 μL of mobile phase (methanol: water = 70:30, v/v) and centrifuged at 12,000 rpm for 10 min, and a 20 μL aliquot injected into HPLC/MS system (Shimadzu HPLC system/ AB SCIEX Triple Quad™ 5500 System) with MultiQuant 2.1.1software for analysis. Standard calibration curves were prepared by adding 10 μL of dilutions of working solution of SM1044 to the control liver homogenate of 90 μL, respectively.

### Statistical analysis

Data are presented as the mean ± standard deviation (SD) of triplicate or three independent experiments, and all statistical analysis were performed with PRISM 5.0 (GraphPad Software, Inc, La Jolla, CA, USA). In Tables 1 and 2, multiple comparisons of IC_50_ among pairs of curves was performed by log transforming IC_50_ and standard error and then analysed by one-way ANOVA (nonparametric) followed by post-test of Bonferroni’s Multiple Comparison Test. In Fig. 4, repeated measures ANOVA followed by post-test of Dunnett’s Comparison test was used to analyse the relative levels of H_2_O_2_ and ONOO^−^ at various time-points compared with the control. In Fig. 5, two-tailed unpaired *t-*test of was used to compare the viable cell inhibition rate in the absence with presence of scavenger after SM1044 treatment. In Fig. 6, comparison of tumour growth inhibition rate among groups was performed using Chi-square test. Multiple group comparisons in other figures were analysed by one-way ANOVA followed by post-test of Dunnett’s or Bonferroni’s multiple comparison test. Comparison of body weights before and after treatment was analysed via paired *t*-test. Data were considered statistically significant at *P < 0.05 and **P < 0.01.

## Supplementary information

Supplementary information
