# Supplementary material for: Novel dihydroartemisinin dimer containing nitrogen atoms inhibits growth of endometrial cancer cells and may correlate with increasing intracellular peroxynitrite
Source: Sci Rep. 2019 Oct 29;9:15528. doi: 10.1038/s41598-019-52108-6 (PMC6820742; doi:10.1038/s41598-019-52108-6)
Supplement: Supplementary file 1 — Supplementary information [file 41598_2019_52108_MOESM1_ESM.pdf]

Novel dihydroartemisinin dimer containing nitrogen atoms inhibits growth of endometrial cancer cells and may correlate with increasing intracellular peroxynitrite

Yan Zhu *, Christian Klausen, Jieyun Zhou, Xiangjie Guo, Yu Zhang, Hua Zhu, Zhao Li,
Jung-Chien Cheng, Shuwu Xie, Wenjie Yang, Ying Li, Peter C.K. Leung*

V (mm^3) =1/2×a×b^2=1/2*major axis*minor axis*minor axis   Double figures mean there are two separate tumours and the volume is calculated by adding both together

		0 (Before dosing)				Dosing started --1st				2nd			
**Group**	**NO.**	**Body Weight(g)**	**Major axis(mm)**	**Minor axis(mm)**	**Volum (mm3)**	**B.W(g)**	**Major axis(mm)**	**Minor axis(mm)**	**Volum (mm3)**	**B.W(g)**	**Major axis(mm)**	**Minor axis(mm)**	**Volum (mm3)**
**control**	**37**	**23.5**	7.33	5.46	109.26	23.0	7.64	6.50	161.40	23.1	8.36	6.60	182.08
	**56**	20.7	6.27	5.49	94.49	21.4	6.93	6.09	128.51	21.9	7.99	6.51	169.31
	**66**	22.0	5.72/6.48	4.05/4.63	116.36	22.6	6.45/6.91	4.85/5.13	166.78	23.4	7.00/7.42	4.75/6.42	231.88
	**79**	23.2	8.37	5.98	149.66	23.6	9.42	6.29	186.35	25.3	10.89	7.93	342.41
	**91**	22.3	8.42	4.58	88.31	23.3	9.08	5.06	116.24	23.7	10.21	5.41	149.41
	**120**	20.9	7.23	4.98	89.65	21.3	7.76	5.43	114.40	21.7	8.17	6.45	169.95
	**5**	22.1	8.37	5.81	141.27	22.6	9.11	5.51	138.29	23.5	9.59/5.30	7.05/3.07	263.30
	**77**	23.8	7.49	5.11	97.79	22.7	10.57	5.45	156.98	23.0	10.94	6.04	199.55
	**ava**				110.85				146.12				213.49
	**SD**				23.48				25.82				63.89
**carbo 3mg/kg**	**119**	21.1	8.10	6.71	182.35	21.4	8.42	6.25	164.45	20.7	8.08	5.83	137.32
	**36**	21.4	6.97	5.00	87.13	21.6	7.44	4.85	87.50	22.2	7.47	5.3	104.92
	**60**	22.9	6.28/3.71	4.44/3.51	84.75	23.5	6.38/3.59	4.80/2.45	84.27	23.3	7.17/4.19	4.89/3.65	113.63
	**35**	21.1	9.82	4.92	118.85	21.3	10.97	6.37	222.56	22.9	11.60	6.54	248.08
	**111**	20.5	6.91	5.05	88.11	21.2	8.19	5.62	129.34	21.3	9.01	5.50	136.28
	**75**	22.8	7.03/3.85	4.77/2.84	95.50	22.3	6.73/3.85	4.70/3.85	102.86	23.3	8.27/3.77	5.60/3.36	150.95
	**84**	21.1	6.97	4.49	70.26	22.5	7.67	5.64	121.99	22.4	8.55	6.43	176.75
	**80**	24.6	5.66	5.13	74.48	24.6	6.28	5.95	111.16	24.5	7.09	5.94	125.08
	**ava**				100.18				128.02				149.12
	**SD**				36.32				45.95				45.75
**1044 2.5mg/kg**	**4**	22.1	7.00	5.75	115.72	22.1	8.42	6.60	183.39	21.5	8.73	4.21	77.37
	**32**	24.3	6.27	4.94	76.51	24.8	7.24	6.14	136.47	24.5	7.91	6.56	170.20
	**38**	21.8	5.40/3.81	4.33/3.18	69.88	22.7	6.87	5.44	101.65	21.5	9.56	6.54	204.45
	**42**	23.3	5.04/6.18	4.93/2.70	83.78	23.9	7.36/6.27	5.71/3.22	152.49	24.0	8.02/7.39	6.47/4.12	230.58
	**58**	22.0	6.49/3.94/5.97	3.47/3.55/4.07	113.35	22.8	7.69/9.51	5.65/4.39	214.38	22.6	15.30	5.99	274.48
	**94**	23.7	7.20	4.61	76.51	24.8	8.75	5.62	138.18	23.2	9.15	6.47	191.51
	**ava**				89.29				154.43				191.43
	**SD**				20.06				39.52				66.43
**1044 5mg/kg**	**46**	22.1	6.58	5.44	97.36	21.1	8.80	4.53	90.29	17.9	6.60	6.12	123.60
	**67**	23.6	7.31	5.05	93.21	22.9	7.94	6.07	146.27	22.0	8.04	5.89	139.46
	**83**	22.9	5.54/4.09	5.34/3.08	98.39	23.3	7.74	6.02	140.25	20.1	6.82	5.45	101.29
	**107**	24.1	7.51	5.39	109.09	24.2	7.95	6.01	143.58	22.8	8.73	6.52	185.56
	**116**	23.3	6.99	5.63	110.78	23.4	7.28	5.14	96.17	20.5	8.25	5.51	125.24
	**118**	22.6	7.69	5.52	117.16	22.2	7.89	5.66	126.38	19.5	8.51	5.21	115.50
	**ava**				104.33				123.82				131.77
	**SD**				9.34				24.74				29.17

V (mm^3) =1/2×a×b^2=1/2*major axis*minor axis*minor axis

		3rd				4th				5th				6th			
**Group**	**NO.**	**B.W(g)**	**Major axis(mm)**	**Minor axis(mm)**	**Volum (mm3)**	**B.W(g)**	**Major axis(mm)**	**Minor axis(mm)**	**Volum (mm3)**	**B.W (g)**	**Major axis(mm)**	**Minor axis (mm)**	**Volum (mm3)**	**B.W(g)**	**Major axis(mm)**	**Minor axis(mm)**	**Volum (mm3)**
	**37**	23.3	9.53	7.83	292.14	23.9	11.21	7.77	338.39	23.3	11.57	9.59	532.04	25.1	12.25	9.70	576.30
	**56**	22.5	8.66	6.54	185.20	22.9	9.73	6.65	215.14	23.0	11.04	8.05	357.71	23.2	12.00	8.29	412.34
	**66**	23.9	8.10/7.52	5.68/5.69	252.40	23.5	8.73/7.58	5.98/5.33	263.76	23.7	13.53	9.16	567.62	24.6	15.20	10.23	795.36
**control**	**79**	24.7	12.16	8.12	400.88	26.0	13.01	8.73	495.76	25.5	14.93	9.42	662.42	25.4	17.61	7.63	512.60
	**91**	24.7	11.35	6.27	223.10	23.4	12.78	6.04	233.12	22.7	13.17	7.29	349.95	23.2	15.78	8.17	526.65
	**120**	21.6	8.22	6.27	161.58	21.1	9.46	6.95	228.47	20.8	9.85	7.73	294.28	20.5	10.81	9.23	460.47
	**5**	23.8	11.08/5.87	7.58/3.00	344.72	23.2	12.03	7.90	375.40	22.9	13.94	9.25	596.37	23.4	14.46/6.41	8.72/4.46	613.51
	**77**	21.5	10.87	5.80	182.83	24.0	11.76	6.62	257.69	23.6	11.30	6.48	237.25	23.5	12.60	6.54	269.46
	**ava**				255.36				300.97				449.70				520.84
	**SD**				84.97				96.64				158.18				153.87
	**119**	21.4	9.37	6.46	195.51	21.9	9.65	7.01	237.10	21.5	12.10	7.19	312.76	21.8	12.93	8.27	442.16
	**36**	22.6	9.28	6.4	190.05	22.2	9.57	6.85	224.52	22.6	11.45	8.11	376.55	22.8	12.25	9.00	496.13
	**60**	23.5	8.05/5.93	6.55/4.64	236.52	23.3	6.71/8.70	4.86/6.75	277.44	23.8	12.50	7.93	393.03	23.6	13.18	9.45	588.50
**carbo 3mg/kg**	**35**	23.2	12.93	6.41	265.63	22.7	13.26	7.54	376.93	21.9	14.09	10.08	715.82	22.1	15.65	10.58	875.90
	**111**	21.0	9.67	6.74	219.64	21.1	10.15	6.76	231.92	20.8	10.84	6.76	247.68	20.4	11.38	8.00	364.16
	**75**	23.7	8.93/4.02	6.13/3.75	196.05	23.7	9.73/4.74	6.62/3.92	249.62	23.4	10.04/5.44	7.54/4.21	333.60	23.9	11.08/7.23	7.70/5.52	438.62
	**84**	21.9	8.78	6.38	178.69	23.5	9.31	6.43	192.46	22.6	10.82	6.69	242.13	22.6	11.35	8.15	376.95
	**80**	24.3	7.41	6.62	162.37	25.0	7.81	9.49	351.68	24.7	8.90	7.23	232.61	25.4	9.69	7.42	266.75
	**ava**				205.56				267.71				356.77				481.15
	**SD**				33.35				64.53				157.46				185.71
	**4**	21.8	10.82	7.19	279.68	21.0	9.78	7.49	274.33	21.2	11.86	8.94	473.95	22.3	11.64	9.52	527.47
	**32**	23.8	8.09	7.06	201.62	23.9	9.07	6.60	197.54	23.8	9.63	7.57	275.92	24.5	10.73	6.59	232.99
**1044 2.5mg/kg**	**38**	21.8	10.39	6.83	242.34	21.7	10.27	6.64	226.40	21.9	11.01	7.99	351.44	23.0	11.46	7.60	330.96
	**42**	23.3	10.48	7.34	282.31	23.2	8.47/8.71	8.33/4.30	374.39	23.1	8.94/9.09	8.59/5.76	480.62	24.2	12.73	9.32	552.88
	**58**	22.4	9.06/11.07	4.67/6.47	330.49	22.3	12.08/10.18	7.23/5.34	460.87	22.7	11.45/12.40	7.13/8.21	708.95	23.7	17.10	10.62	964.31
	**94**	23.4	10.21	7.18	263.28	22.9	10.39	8.08	339.16	23.0	11.35	7.71	337.35	24.1	11.27	8.41	398.55
	**ava**				266.62				312.12				438.04				501.19
	**SD**				43.18				98.63				155.19				256.62
	**46**	19.4	7.34	6.76	167.71	19.8	8.29	7.53	235.03	20.8	8.53	7.91	266.85	20.9	9.35	7.90	291.77
	**67**	22.1	9.20	6.57	198.56	22.2	9.34	6.45	194.28	23.9	9.61	6.65	212.49	23.9	9.72	7.63	282.93
**1044 5mg/kg**	**83**	23.6	8.27	6.70	185.62	22.9	7.87	7.51	221.93	23.3	9.83	7.08	246.37	23.2	9.66/5.56	7.41/4.56	323.01
	**107**	24.5	9.53	6.26	186.73	24.4	8.83	7.32	236.57	25.2	10.32	7.65	301.98	25.2	11.67	8.26	398.11
	**116**	24.5	8.28	6.07	152.54	23.8	10.20	6.55	218.80	24.5	10.38	7.05	257.96	23.8	12.49	7.36	338.29
	**118**	21.3	8.32	6.07	153.27	20.6	9.56	6.96	231.55	21.5	10.13	7.60	292.55	21.0	11.41	7.91	356.95
	**ava**				174.07				223.03				263.03				331.84
	**SD**				19.13				15.78				32.44				42.75

V (mm^3) $=1/2\times a\times b^2$=1/2*major axis*minor axis*minor axis

		7th				8th				9th				10th			
**Group**	**NO.**	**B.W(g)**	**Major axis(mm)**	**Minor axis(mm)**	**Volum (mm3)**	**B.W(g)**	**Major axis (mm)**	**Minor axis(mm)**	**Volum (mm3)**	**B.W(g)**	**Major axis(mm)**	**Minor axis(mm)**	**Volum (mm3)**	**B.W(g)**	**Major axis(mm)**	**Minor axis(mm)**	**Volum (mm3)**
**control**	**37**	24.5	13.26	9.57	607.21	23.7	13.15	10.12	673.37	23.6	14.27	10.26	751.08	23.4	15.27	10.97	918.80
	**56**	23.4	12.67	8.28	434.32	23.0	13.52	9.28	582.16	22.8	14.57	9.70	685.45	23.2	15.39	10.08	781.86
	**66**	25.1	16.59	10.86	978.31	24.0	16.72	12.07	1217.93	24.7	17.84	12.70	1438.71	24.6	17.87	13.31	1582.89
	**79**	24.9	18.30	11.43	1195.40	26.0	19.81	11.85	1390.88	25.7	20.81	12.46	1615.39	25.9	21.72	12.73	1759.89
	**91**	22.3	16.09	8.80	623.00	23.9	17.63	9.33	767.34	24.1	18.72	9.94	924.80	23.4	19.65	10.99	1186.66
	**120**	21.0	10.72	9.59	492.95	19.8	11.12	8.87	437.44	21.0	12.43	10.63	702.28	20.8	12.35	10.43	671.75
	**5**	24.0	15.63	9.62	723.23	24.4	18.00	9.11	746.93	24.7	18.06	9.76	860.18	24.4	18.67	9.10	773.03
	**77**	24.1	14.43	7.79	437.84	24.5	14.11	8.04	456.05	25.1	14.67	8.33	508.97	24.9	15.06	9.25	644.29
	**ava**				686.53				784.01				935.86				1039.90
	**SD**				272.21				346.18				388.06				427.50
**carbo 3mg/kg**	**119**	22.8	14.35	8.42	508.68	23.5	14.93	10.46	816.76	23.3	15.36	10.20	799.03	23.3	16.99	10.82	994.53
	**36**	22.4	13.33	8.94	532.69	22.0	14.86	10.27	783.66	22.2	15.95	11.18	996.81	21.8	16.07	11.33	1031.44
	**60**	23.4	13.79	9.01	559.74	24.2	14.48	10.39	781.57	23.3	16.20	11.54	1078.69	23.3	16.49	12.11	1209.15
	**35**	21.7	15.78	11.00	954.69	23.0	15.96	8.88	629.26	22.6	16.75	10.17	866.22	22.2	16.62	10.37	893.63
	**111**	19.1	11.93	8.51	431.99	19.7	12.64	8.81	490.53	19.5	14.69	9.01	596.27	19.6	15.08	9.18	635.41
	**75**	23.4	11.01/6.87	8.60/5.74	520.32	23.6	11.67/7.70	8.62/5.71	559.09	23.4	11.82/8.09	9.39/6.59	696.76	23.1	13.03/8.83	9.95/6.86	852.77
	**84**	21.2	12.74	8.58	468.94	20.7	12.99	8.16	432.47	21.9	13.36	8.57	490.61	21.8	13.69	9.69	642.72
	**80**	25.6	9.10	7.58	261.43	25.7	8.91	7.08	223.31	26.1	9.80	7.82	299.65	24.8	9.40	7.88	291.84
	**ava**				529.81				589.58				728.00				818.94
	**SD**				195.40				206.08				261.20				287.24
**1044 2.5mg/kg**	**4**	22.0	12.44	9.88	607.16	21.6	12.70	9.60	585.22	21.9	13.65	5.93	240.00	22.3	13.81	9.96	684.99
	**32**	25.0	10.18	8.40	359.15	23.9	8.75	8.64	326.59	23.9	9.01	8.66	337.86	23.9	10.08	9.61	465.45
	**38**	22.9	10.73	7.69	317.27	22.5	11.33	7.71	336.75	22.5	11.41	7.56	326.06	22.2	11.26	7.75	338.15
	**42**	25.0	11.83/7.58	6.47/7.01	433.85	24.1	11.67/7.78	6.87/6.99	465.46	24.3	13.05/6.88	7.76/6.65	545.06	24.2	13.48/8.12	7.23/7.12	558.14
	**58**	23.8	16.92	12.93	1414.38	22.6	16.63	13.24	1457.60	23.7	16.86	12.55	1327.75	23.5	17.33	13.17	1502.93
	**94**	23.8	11.39	8.68	429.07	23.2	13.29	9.69	623.94	23.4	13.53	9.47	606.69	22.5	12.29	10.14	631.83
	**ava**				593.48				632.59				563.90				696.92
	**SD**				414.19				422.39				399.56				413.66
**1044 5mg/kg**	**46**	21.3	8.95	7.62	259.84	22.5	9.91	9.21	420.30	22.0	11.16	8.77	429.17	22.3	11.54	9.02	469.45
	**67**	24.1	10.66	7.92	334.33	25.4	10.20	8.19	342.09	23.9	11.32	7.82	346.12	23.5	10.98	8.07	357.54
	**83**	23.4	10.84	7.93	340.84	24.0	11.17	8.00	357.44	23.2	10.86/6.70	8.15/6.48	501.34	23.5	12.54	9.65	583.88
	**107**	25.2	12.12	7.50	340.88	26.0	13.77	10.15	709.31	25.9	14.50	9.67	677.94	26.0	15.22	10.44	829.44
	**116**	24.3	10.81	7.29	287.24	25.1	12.52	7.36	339.10	23.8	13.36	7.36	361.85	24.9	13.74	8.11	451.97
	**118**	22.2	11.35	7.11	286.88	23.1	11.58	7.74	346.87	21.5	12.52	8.27	428.14	22.5	14.38	9.29	620.53
	**ava**				308.33				419.18				457.43				552.13
	**SD**				34.78				145.31				121.44				165.72

V (mm^3) $=1/2\times a\times b^2$=1/2*major axis*minor axis*minor axis

		11th				12th (the last time)				Anatomy				
**Group**	**NO.**	**B.W(g)**	**Major axis(mm)**	**Minor axis(mm)**	**Volum (mm3)**	**B.W(g)**	**Major axis(mm)**	**Minor axis(mm)**	**Volum (mm3)**	**weight of tumor(g)**	**liver (g)**	**Kidney (g)**	**Coefficient of liver**	**Coefficient of kidney**
**control**	**37**	23.0	15.47	10.97	930.84	24.3	15.43	12.26	1159.62	0.727	1.511	0.315	0.0622	0.0130
	**56**	23.8	15.73	10.77	912.28	23.6	16.35	10.83	958.84	0.727	1.511	0.315	0.0640	0.0133
	**66**	25.2	18.35	14.03	1806.02	24.5	18.82	14.82	2066.74	1.252	1.638	0.369	0.0669	0.0151
	**79**	26.5	22.14	14.43	2305.05	26.7	23.32	13.65	2172.52	1.775	1.778	0.339	0.0666	0.0127
	**91**	24.0	21.21	11.63	1434.40	23.9	22.21	12.08	1620.51	1.701	1.392	0.326	0.0582	0.0136
	**120**	21.6	12.61	10.91	750.47	21.5	12.78/9.07	8.44/9.06	827.43	0.804	1.184	0.263	0.0551	0.0122
	**5**	21.4	18.70	9.82	901.64	25.2	18.55	10.72	1065.87	1.044	1.486	0.313	0.0590	0.0124
	**77**	25.1	15.02	9.35	656.54	24.9	15.03	8.04	485.78	0.542	1.560	0.356	0.0627	0.0143
	**ava**				1212.16				1294.66	1.07	1.51	0.32	0.062	0.013
	**SD**				583.56				601.06	0.47	0.17	0.03	0.004	0.001
**carbo 3mg/kg**	**119**	22.7	16.72	12.67	1342.02	23.0	17.90	11.71	1227.26	0.942	1.091	0.301	0.0474	0.0131
	**36**	23.1	16.00	11.64	1083.92	22.7	16.92	11.87	1191.99	0.942	1.091	0.301	0.0481	0.0133
	**60**	23.7	16.77	12.77	1367.37	22.4	15.99	13.13	1378.31	1.038	1.199	0.326	0.0535	0.0146
	**35**	22.8	16.59	10.13	851.21	22.8	16.00	10.03	804.81	0.885	1.406	0.350	0.0617	0.0154
	**111**	19.8	13.78	9.29	594.64	19.6	14.53	9.97	722.15	0.618	0.985	0.258	0.0503	0.0132
	**75**	23.5	13.74/7.43	10.57/6.61	929.87	22.9	14.35/8.05	10.69/6.73	1002.23	0.990	1.140	0.287	0.0498	0.0125
	**84**	21.4	15.40	10.01	771.54	21.1	15.40	8.56	564.21	0.618	1.168	0.292	0.0554	0.0138
	**80**	25.4	10.32	8.16	343.58	25.1	11.07	8.33	384.07	0.245	1.520	0.366	0.0606	0.0146
	**ava**				910.52				909.38	0.78	1.20	0.31	0.053	0.014
	**SD**				352.42				348.57	0.27	0.18	0.04	0.005	0.001
**1044 2.5mg/kg**	**4**	22.6	14.54	9.79	696.79	23.2	14.36	10.39	775.10	0.512	1.469	0.348	0.0633	0.0150
	**32**	24.4	10.62	9.06	435.86	24.9	10.15	8.56	371.86	0.245	1.826	0.351	0.0733	0.0141
	**38**	23.0	11.55	8.49	416.26	22.6	11.50	7.90	358.86	0.352	1.472	0.339	0.0651	0.0150
	**42**	24.3	13.97/8.04	8.54/7.32	724.83	24.9	14.43/9.23	7.80/8.10	741.75	0.569	1.481	0.330	0.0595	0.0133
	**58**	23.8	17.95	12.93	1500.48	24.5	18.01	14.47	1885.48	1.004	1.557	0.342	0.0636	0.0140
	**94**	22.9	14.24	9.69	668.54	22.3	15.01	10.68	856.04	0.592	1.43	0.321	0.0641	0.0144
	**ava**				740.46				831.51	0.55	1.54	0.34	0.065	0.014
	**SD**				395.69				558.10	0.26	0.15	0.01	0.005	0.001
**1044 5mg/kg**	**46**	21.8	11.94	9.38	525.27	21.1	11.74	9.30	507.70	0.435	1.187	0.307	0.0563	0.0145
	**67**	24.0	10.96	6.00	197.28	23.6	11.30	8.65	422.75	0.365	1.240	0.341	0.0525	0.0144
	**83**	23.5	12.63	10.37	679.10	23.2	12.87	11.05	785.73	0.499	1.420	0.292	0.0612	0.0126
	**107**	26.3	17.23	9.58	790.65	26.0	17.68	10.75	1021.57	1.046	1.715	0.368	0.0660	0.0142
	**116**	25.1	14.79	9.22	628.64	23.8	16.06	8.95	643.22	0.568	1.677	0.362	0.0705	0.0152
	**118**	23.0	13.99	9.47	627.32	22.6	14.83	8.29	509.59	0.451	1.366	0.309	0.0604	0.0137
	**ava**				574.71				648.43	0.56	1.43	0.33	0.061	0.014
	**SD**				204.01				222.77	0.25	0.22	0.03	0.006	0.001
